# A *Brassica napus* Lipase Locates at the Membrane Contact Sites Involved in Chloroplast Development

**DOI:** 10.1371/journal.pone.0026831

**Published:** 2011-10-26

**Authors:** Xiaoli Tan, Qiuye Wang, Baoxia Tian, Henan Zhang, Daoli Lu, Jia Zhou

**Affiliations:** Institute of Life Sciences, Jiangsu University, Zhenjiang, Jiangsu, People's Republic of China; University of Hyderabad, India

## Abstract

**Background:**

Fatty acids synthesized in chloroplast are transported to endoplasmic reticulum (ER) for triacylglycerols (TAGs) resembling. The development of chloroplast also requires lipids trafficking from ER to chloroplast. The membrane contact sites (MCSs) between ER and chloroplast has been demonstrated to be involved for the trafficking of lipids and proteins. Lipids trafficking between ER and chloroplast is often accompanied by lipids interconversion. However, it is rarely known how lipids interconversion happens during their trafficking.

**Methodology/Principal Findings:**

We cloned a lipase gene from *Brassica napus* L., designated as *BnCLIP1*. Green fluorescence protein (GFP)-tagged BnCLIP1 was shown to locate at the MCSs between ER and chloroplasts in tobacco leaves. Heterogeneous expression of BnCLIP1 in *Saccharomyces cerevisiae (pep4*) reduced the total amount of fatty acid. Gas chromatography-mass spectrometry (GC-MS) analysis revealed that the truncated BnCLIP1 had a substrate preference for C16:0 lipids in *Saccharomyces cerevisiae (pep4*). To probe the physiological function of BnCLIP1, two *Brassica napus* lines with different oil-content were introduced to investigate the transcript patterns of *BnCLIP1* during seed development. Intriguingly, the transcript level of *BnCLIP1* was found to be immediately up-regulated during the natural seed senescence of both lines; the transcription response of *BnCLIP1* in the high oil-content seeds was faster than the lower ones, suggesting a potential role of BnCLIP1 in affecting seed oil synthesis via regulating chloroplast integrity. Further researches showed that chemical disruption of leaf chloroplast also activated the transcription of *BnCLIP1*.

**Conclusions/Significance:**

The findings of this study show that *BnCLIP1* encodes a lipase, localizes at the MCSs and involves in chloroplast development.

## Introduction

Lipase, a member of the super-family of hydrolytic/lipolytic enzymes, contains a highly conserved catalytic triad formed by — serine, aspartic acid, and histidine [1]. The Ser residue is the most important residue for the fatty acid-deesterifying activity [2]. Lipase can be detected in the roots, inflorescence stems, flowers, siliques, and leaves of plants, oil bodies, plastoglobuli, glyoxysomes, or microsomal fractions of seed extracts, depending on the plant species[3], [4]. It is known that chloroplast plastoglobuli of senescing leaves are bigger and more abundant than those in the chloroplast of non-senescing leaves [4], [5]. Recently, a plastid TAG lipase AAD24845 was reported to be involved in the maintenance of the structural integrity of chloroplasts, probably by reutilizing the fatty acids of degraded plastid TAGs [4].

In plants, lipids are usually stored in the form of triacylglycerols (TAGs) [6]. TAGs are often aggregated into oil bodies in seeds and plastoglobuli in chloroplasts [7]. Storage plastids containing neutral lipids and TAGs have also been identified in some nonphotosynthetic tissues [8], [9]. Lipid bodies were considered to be located in the mesophyll tissue of leaves [10], [11] until the middle of the last century. In leaves, lipid bodies containing TAGs were mainly located in the chloroplast, and the fatty acid composition of the chloroplast TAGs was very similar to that of the seed TAGs [7], [12].

Lipids synthesized from the ER are transported to their target membranes via the MCSs outside the secretory pathway [13]. In plant, the physical associations between ER membrane and non-green plastids have been demonstrated in several tissues [14], [15], [16], [17]. Recently, the MCSs between ER and chloroplasts have also been observed in Arabidopsis by Mats X. Andersson et al. [18], [19]. The lipid trafficking at the MCSs between ER and chloroplasts was also intensively investigated [20]. Xu et al. revealed that the TRIGALACTOSYLDIACYLGLYCEROL (TGD) proteins are involved in unidirectional lipids transferring from ER to the plastid in Arabidopsis [9], [21], [22], [23], [24], [25]. Phosphate depriving experiments showed that phospholipids can be replaced by DIGALACTOSYLDIACYLGLYCEROL (DGDG) in plasma membranes [26], [27], mitochondria [28] and tonoplasts [26]. It was well known that fatty acids released from plastids can be transformed into phosphatidylcholine in ER and transferred through ER outside envelope membrane contact zones (PLAM) in the form of phosphatidic acid [29], [30], [31]. Howerver, it is still not clear how the lipids are interconverted during trafficking.

In this study, a *Brassica napus* originated lipase designated as BnCLIP1 was shown for the first time to be involved in the lipid interconversion. BnCLIP1 locates at the MCSs between ER and chloroplasts in tobacco leaves. Over expression of the truncated BnCLIP1 reduces the total amount of both neutral lipids and polar lipids, and the truncated BnCLIP1 prefers C16:0 lipids as substrate in *Saccharomyces cerevisiae (pep4)*. Moreover, both chloroplast natural senescence and chemical disruption can activate the transcription expression of *BnCLIP1*.

## Methods

### Plant materials and chemical treatments

Oil seeds of *Brassica napus* cv. Ningyou16 were sterilized and grown on solidified Murashige and Skoog (MS) media as described previously [32]. Roots, stems, leaves, and flowers were harvested from 6-month-old plants. Seeds at 25 days after pollination (DAP), 35 DAP, 45 DAP, and 50 DAP were collected from *Brassica napus* lines EM91 (oil-content: 29.62%) and EM102 (oil-content: 50.59%), and used to analyze the transcription profiles of *BnCLIP1*. A piece of leaf of a 4-week-old plant was soaked in spectinomycin (SPCM) (100 mg/l) for 5 s every day *in vivo*, and 1 week later, another leaf situated near this senescing leaf was picked and sliced into 2 pieces. One piece was soaked in 100 mg/l SPCM for 1 hour, while the other was soaked in ddH_2_O for 1 hour as a control. RNAs were immediately extracted and processed for quantitative real-time polymerase chain reaction (PCR).

*N. benthamiana* seeds were sown in a vermiculite/soil mixture under a 16∶8-h light/dark light condition at a constant temperature of 25°C. After four weeks' growing, the leaves were used for infiltrating Agrobacterium to transiently express exogenous genes.

### *In silico* cloning and 3′ rapid amplification of cDNA end (RACE)

Total RNAs were extracted using Plant RNA Reagent (Invitrogen, CA, USA). The contaminated genomic DNAs in RNA were digested by DNase I (Takara, Japan). The first strand cDNA was synthesized using 2 µg of total RNAs and Moloney-murine leukemia virus (M-MLV) Reverse Transcriptase (Takara, Japan). Synthesized cDNAs were then used for 3′ RACE and diluted 10 times for real-time PCR assay. *Arabidopsis* gene *AT1G06800* was used as the query sequence to search the *Brassica* expressed sequence tags (ESTs) database using the basic local alignment search tool (BLAST) algorithm (http://www.arabidopsis.org/). The BLAST search returned 17 homologous ESTs, and of these, 6 ESTs with the highest identities (GeneBank Acc. Nos. AM389405, AM390098, EV221383, EX098538, EX105251, and EX135634) were used for *in silico* cloning. The 3′ RACE was performed according to the manufacturer's instruction (Takara, Japan). Specific primers for BnCLIP1 3-1 (5′-GCGATCTGAGCATCACGGT-3′) (first PCR) and BnCLIP1 3-2 (5′-GCTGAACAGGACGAAGAAT-3′) (second PCR) were used for Nest PCR.

### Sequence and Phylogenetic analysis

GXSXG lipase-like domains were identified in various lipases by querying the National Center for Biotechnology Information (NCBI) Conserved Domain Database. Homology analysis was performed using ClustalW and Genedoc. Pattern search was performed in the Protein Information Resource (PIR) (http://pir.georgetown.edu/pirwww/index.shtml) network. Subcellular localization prediction was performed using TargetP 1.1 Server (http://www.cbs.dtu.dk/services/TargetP/). Signal peptide prediction was carried out using ChloroP (http://www.cbs.dtu.dk/services/ChloroP-1.1/). A phylogenetic tree was constructed by the neighbor-joining method using molecular evolutionary genetics analysis (MEGA) (version 4.0).

### Crude enzyme activity assay

The predicted signal peptide containing 45 aa was removed during the PCR cloning for generating BnCLIP1 expression constructs. *Eco*RI and *Not*I restriction sites were introduced into the sense primer BnCLIP1-E-F (5′-gaattcATGGCTGTGTCGAGAACC-3′) with an extra “ATG” and the antisense primer BnCLIP1-ORF-R (5′- gcggccgcTGAAGGGTGATGGAGTTG-3′), respectively. The PCR product was ligated into the yeast expression vector pYES2, generating pYES2_BnCLIP1′.

A protease-A-deficient (*pep4*) strain of *S. cerevisiae* was selected as the expression host. Yeast transformation was performed as described previously [33]. *pYES2* transformants, as negative controls, and *pYES2_BnCLP1′* transformants were cultured to log phase in 200 ml of YPD at 30°C. Pellets were washed 4 times with ddH_2_O, and then induced in 40 ml of Dropout base liquid medium with 2% galactose under the same conditions. After 12 hours of incubation, the cells were pelleted and crushed by grinding in liquid nitrogen, and then were suspended in 50 mM sodium phosphate buffer (pH 7.0). The supernatant was used for lipase activity assay, as described by Hong JK et al. [34]. Protein concentration was determined using the Bradford method [35].

### Estimation of intracellular neutral lipids

Sudan black B was used as a marker to determine the content of neutral lipids in *S. cerevisiae*. Cell quantification was performed at λ _600_ nm. The induced yeast cells were stained with 0.1% solution of Sudan black B prepared in 70% ethanol for 10 min and then rinsed in 70% ethanol for more than 3 times. The measurement was performed at λ_ 580_ nm [36].

### Estimation of polar lipids

Total polar lipids of *S. cerevisiae* were extracted using chloroform/methanol, dried by rotary evaporation at 30°C, and then dissolved in chloroform. The polar lipid extract was examined by two-dimensional thin layer chromatography (2D-TLC) on aluminum-backed plates of silica gel 60 F254 (Merck 5554), as described previously [37].

### Analysis of fatty acids

To determine the total fatty acid composition of yeast cells, fatty acid methyl esters from the transformants cultured in Dropout medium were prepared as described previously [38]. The fatty acyl methyl esters were analyzed by gas chromatography (GC) using methyl heptadecanoate (C17:0) (Sigma. USA) as an internal standard. GC analysis was performed on a HP5890 gas chromatograph equipped with a BPX-70 (30 m×0.25 mm) chromatography column. The initial column temperature was 140°C that was held for 10 min. and then raised at 4°C/min until it reached 240°C and held for another 10 min.

### Agrobacterium-mediated transient expression and subcelluar localization

For GFP fusions driven by the cauliflower mosaic virus 35S promoter, the full-length *BnCLIP1* fragment was cloned into vector pK7FWG2.0 [39] via the Gateway recombination system (Invitrogen). The subcellular localization of BnCLIP1 were investigated by co-expression with ER marker fused with YFP and plasma membrane marker fused with RFP [40], respectively, which were obtained from the Arabidopsis Biological Resource Center. Agrobacterium (GV3101)-mediated transient expression assays were carried out according to previous report [41]. A small part of transient expressed leaves were cut off and examined by laser-scanning confocal microscopy using an Olympus (Tokyo, Japan) confocal laser scanning microscope (model FV1000). Excitation and emission wave-lengths for GFP, RFP, YFP, and chloroplast autofluorescence were 488/510–540, 543/581–651, 543/581–651, and 633/661, respectively.

### Quantitative real-time PCR analysis

Quantitative real-time PCR was performed using SYBR® Premix Ex TaqTM II (Takara, Japan), and the amplified products were detected using MX3000P (Stratagene, USA). Gene specific primers for BnCLIP1 3-1 (5′-GCGATCTGAGCATCACGGT-3′) and BnCLIP1-R (5′-AAGGGTGATGGAGTTGGGTG-3′) were used for this experiment. The resultant cDNAs were subjected to 45 cycles of amplification under the following conditions: 95°C denaturing for 1 min, 57°C annealing for 30 s, and 72°C extension for 15 s. The transcription level of leaf pieces on 6-day-old seedlings incubated in water for 1 h was considered as 1.

### Chlorophyll content assay

Chlorophyll was extracted from the seeds crushed in liquid nitrogen using 5 ml of 80% acetone. Seed fragments were separated by centrifugation at 1,200 rpm for 2 min. Chlorophyll *a* and *b* were detected at 663 nm and 645 nm, respectively. The chlorophyll content was calculated according to the MacKinney's specific absorption coefficients [42].

## Results

### Gene cloning and analysis

**Figure 1:**
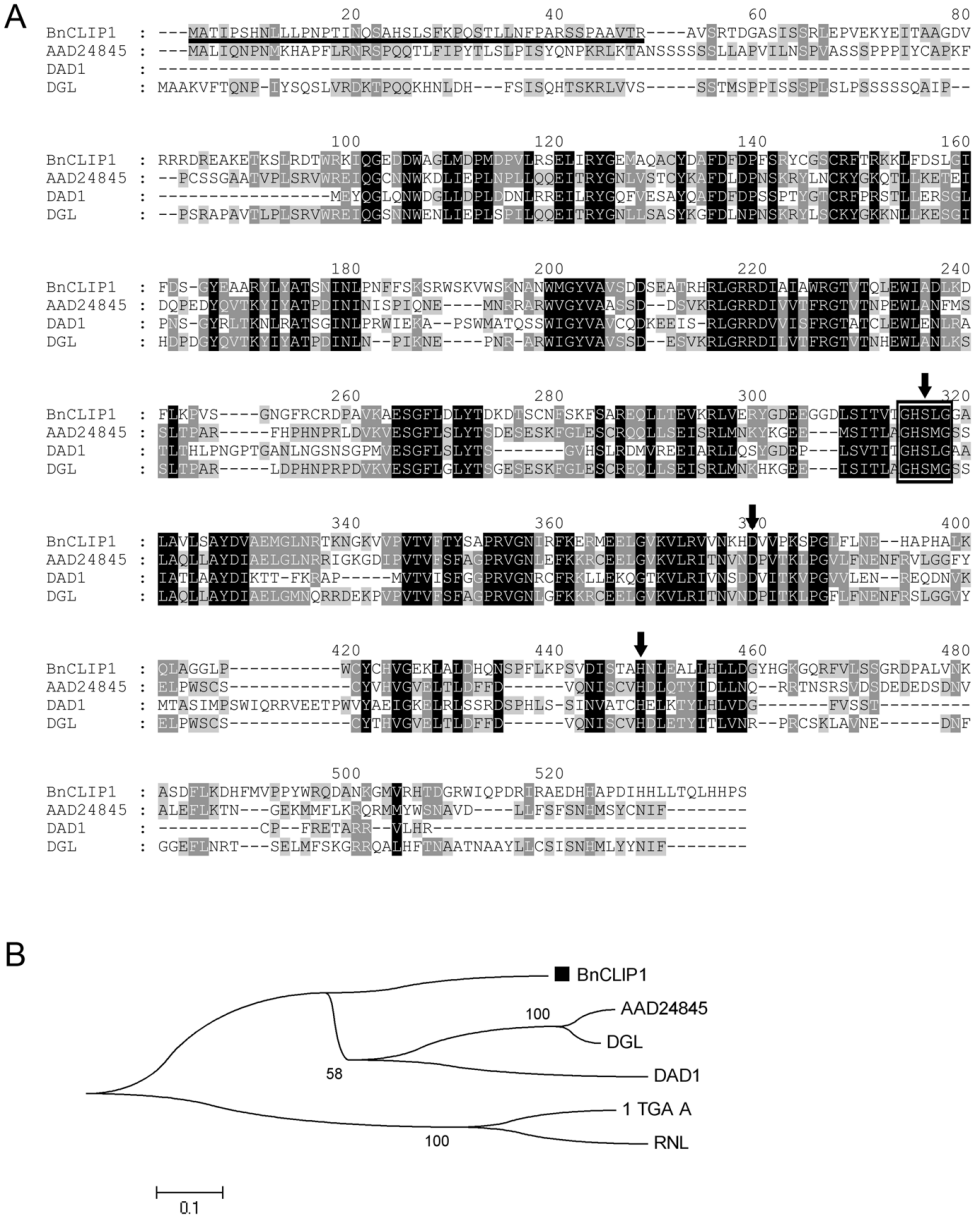
Sequence analysis of BnCLIP1. (A) Alignment of BnCLIP1. The predicted plastid transit peptide is underlined, and the GxSxG lipase motif is marked with a box. The three strictly conserved amino acids Ser^304^, Asp^367^, and His^423^ are marked with dark arrows forming the putative catalytic triad. The BnCLIP1 sequence was aligned against three chloroplast-localized lipases AAD24845, DAD1, and DGL (GenBank Acc. Nos. AAD24845, NP_182008, and ACA48222), respectively. (B) Phylogenetic analysis. Phylogenetic tree was constructed using the sequences of BnCLIP1, AAD24845, DAD1, and DGL, and microorganism lipases 1TGL_A and RNL (GenBank Acc. Nos. 1TGL_A and P61871, respectively). BnCLIP1 is indicated by a black block.

Gene cloning was performed as described in “Materials and methods.” The TargetP and ChloroP programs predicted that the deduced protein is most likely a chloroplast-targeted protein with a putative plastid transit peptide of 45 amino acids at the N-terminus (Fig. 1A), the cleavage of which would produce a mature protein of approximately 53 kD. Sequence analysis demonstrated that the deduced protein contains the GxSxG lipase motif, which is a common feature of lipases (Fig. 1A), and shares a conserved putative catalytic triad “SDH” with three chloroplast-localized lipases (AAD24845 [4], DAD1 [43], DGL [44] ) (Fig. 1A). Ser**^304^**, Asp**^367^**, and His**^423^** form the putative catalytic triad SDH. This gene encoded a chloroplast-targeted lipase thus was designated as *BnCLIP1*, and the gene sequence was submitted to GenBank (GenBank Acc. No. FJ461591)

Phylogenetic analysis was based on the similarity of the conserved domain sequence between BnCLIP1 and the three chloroplast-localized lipases and two microorganism lipases (1TGL_A and RNL). AAD24845 was reported to have a TAG lipase function [4], while DAD1[43] and DGL [44] were proved to be galactolipases with weak phospholipase A1 activity. BnCLIP1 was classified into a new branch that was parallel to the branch containing AAD24845, DAD1, and DGL (Fig. 1B). Sequence similarity analysis showed that BnCLIP1 has 32.4%, 29.0%, and 32.2% sequence identity with AAD24845, DAD1, and DGL, respectively. BnCLIP1 was considered possibly to be a multifunctional lipase.

### The effects of heterogeneously expressed BnCLIP1 on the lipid composition of *S. cerevisiae*

To enhance the enzyme activity of BnCLIP1, a truncate version of BnCLIP1 (BnCLIP1') without the predicted transit peptide (45 aa) was heterogeneously expressed in *S.cerevisiae*. The total proteins extracted from galactose-induced transformants were used for lipase activity assay, and p-nitrophenyl laurate (C12) was used as the substrate. BnCLIP1′ had no significant lipase activity towards p-nitrophenyl laurate (C12) (p>0.05; data not show).

In plant leaves, lipid bodies are mainly located in the chloroplast. The fatty acid composition of chloroplast TAGs is very similar to that of the TAGs found in seeds [7], [12]. Although the composition of TAGs might vary among different tissues and organs, all TAGs share the major components, i.e., TAGs 16∶0, 18∶0, and 18∶1 [12], [45]. Fatty acids in *S. cerevisiae* mainly comprise of C16∶0, C16:1, C18:0, and C18:1 [46], which are very similar to the lipid composition of leaf chloroplast. We detected the 4 main fatty acids in the transformants by GC and found that, comparing with the control, the amount of all the 4 fatty acids were reduced in varying degrees. Total amount of fatty acids were decreased by 19.13%, while the fastest metabolized fatty acid C16:0 was decreased by 28.57% (Table 1).

**Table 1:** The fatty acid composition of galactose-induced *S. cerevisiae* (*pep4*).

FA^a^	pYES2^b^	pYES2_BnCLIP1'^b^	ΔFA^c^	FADR^d^ (%)
C16:0	182±2	130±7	52	29
C16:1	471±7	405±27	66	14
C18:0	55±1	48±2	7	13
C18:1	322±8	270±7.5	52	16
Total	1030±18	853±51	197	19

a , fatty acid.

b , fatty acid extracted from dry yeast (ìg/100 mg).

c , reduced fatty acid in pYES2_BnCLIP1' in contrast to pYES2.

d , fatty acid decreasing rate (ΔFA/ pYES2) ×100.

**Figure 2:**
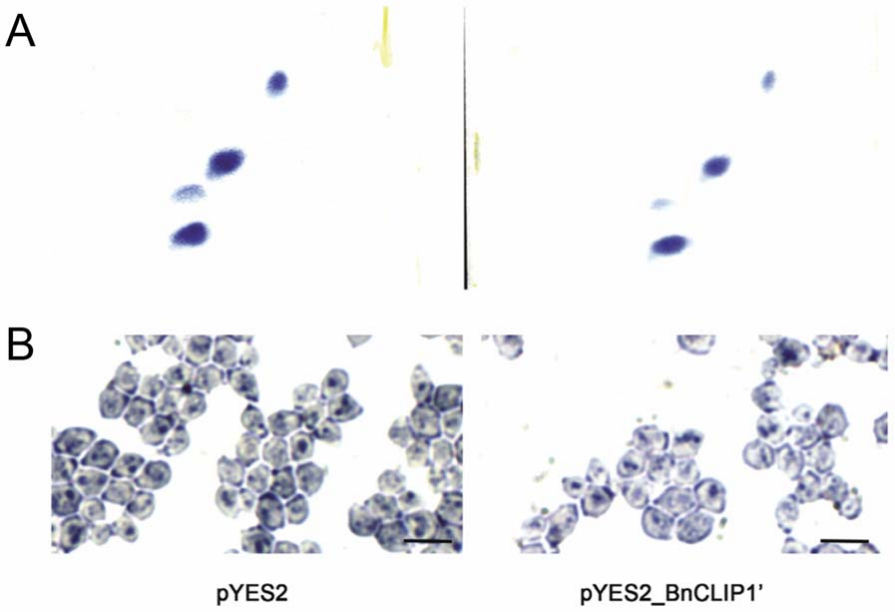
The effects of BnCLIP1 on the lipids and phospholipids of *S.cerevisiae* (*pep4*). (A) Yeast phospholipids analysis with 2D-TLC. (B) Neutral lipid detection of *S. cerevisiae* (*pep4*) by Sudan black B staining. The induced yeast cells were examined under immersion objective. Scale bar  =  5 µm.

The ability of BnCLIP1′ to hydrolyze neutral lipids and polar lipids was detected by Sudan black B staining [36] and 2-D TLC assay, respectively. The amount of both total intracellular neutral lipids and polar lipids were remarkably reduced in the pYES2_BnCLIP1′ harboring strain in contrast to the empty vector harboring strain (Fig. 2A, B).

### Subcellular localization of BnCLIP1 in tobacco leaves and tissue specific expression pattern in *Brassica napus*

**Figure 3:**
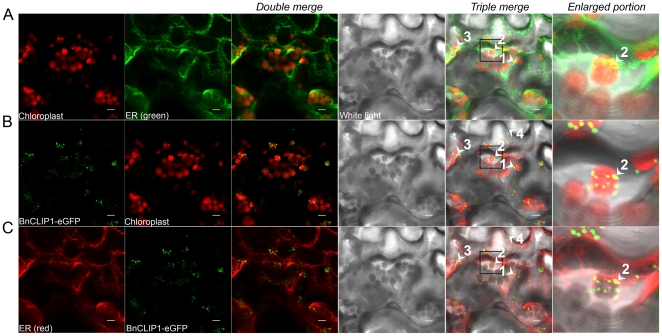
BnCLIP1-eGFP locates at the membrane contact sites between endoplasmic reticulum and chloroplasts in tobacco leaves. (A) The subcellular location of ER relative to chloroplast. (B) The subcellular location of BnCLIP1-eGFP relative to chloroplast. (C) The subcellular location of BnCLIP1-eGFP relative to ER. Chloroplast was shown in red autofluorescence; ER net work marked with yellow fluorescence protein (YFP) was colored red or green for obtaining clear overlapped spots with green or red fluorescence; while the cell counter was shown in the white background. The enlarged portion indicated by black squares was shown at the right side of the triple merged figures. The numbered arrow heads, form number 1 to 3, indicate the triple overlaying points. The arrow head 4 indicates the scattered GFP signal. Scale bars:10ìm.

**Figure 4:**
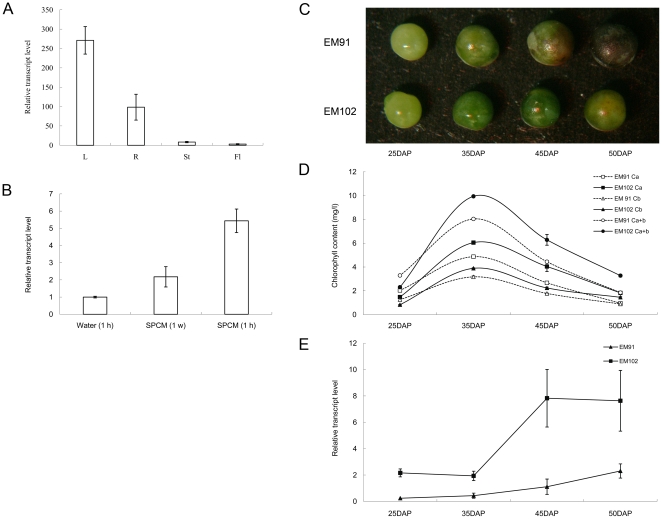
The transcript profiles of *BnCLIP1*during natural chloroplast senescence and chemical induced chloroplast disruption. (A) Organ-specific expression of *BnCLIPI* in the leaves (L), roots (R), stems (St), and flowers (Fl). (B) Expression profile of *BnCLIP1* in the leaves treated with SPCM. “Water (1 h)” represents the control, while the half piece of the control leaf that was treated with 100 mg/l SPCM for 1 hour has been designated as “SPCM (1 h).” “SPCM (1 w)” refers to the leaf of the same age soaked in 100 mg/l SPCM for 5 s everyday for 1 week. (C) EM91 (low oil-content) and EM102 (high oil-content) seeds. (D) Chlorophyll content of developing EM102 and EM91 seeds. (E) Expression profiles of *BnCLIP1* in EM102 and EM91 seeds at 25 DAP, 35 DAP, 45 DAP, and 50 DAP. DAP represents “day after pollination.” *BnActin* was used as an internal control.

To experimentally verify the predicted subcellular location of BnCLIP1 (Fig. 1A), we fused a green fluorescent protein (GFP) to the C-terminal of BnCLIP1 to generate the plant expressing vector under the control of CaMV35S promoter, and then infiltrated the tobacco leaves with it to transiently express the BnCLIP1-eGFP. The subcellular localization of BnCLIP1-eGFP was examined in crude tobacco leaves by confocal laser-scanning microscopy. In figure 3, the yellow fluorescence spots were generated by the double overlapping of red fluorescence and green fluorescence. When the fluorescence for ER and chloroplast were overlapped and further merged with the white light background slide, more yellow fluorescence spots were clearly observed, which indicated the physical interactions between ER and chloroplast (Fig. 3A), chloroplast and BnCLIP1 (Fig. 3B), ER and BnCLIP1 (Fig. 3C), respectively. The triple-interacted sites among ER, chloroplast, and BnCLIP1 were exampled by the numbered arrows heads, from number 1 to number 3, which demonstrated that BnCLIP1 is located at the MCSs between ER and chloroplasts (Fig. 3). Besides, we could also observe some other GFP signals scattering around the ER and plasma membrane, such as arrow 4 in figure 3, and arrow 5 in figure S1 (Fig. 3 and Fig. S1). We thus examined the possibility of BnCLIP1 locating at cytoplasma membrane (Fig. S1). Although some GFP singles distributed near the cytoplasma membrane or ER, we failed to observe any overlapped fluorescent signal (Fig 3 and Fig. S1). Accordingly, tissue specific expression analysis showed that *BnCLIP1* can be detected in both photosynthetic tissues, such as leaf, and non-photosynthetic tissues, such as flower and root (Fig.4A) which was similar to previous report [4]. And roots and other non-photosynthetic tissues were also known to contain neutral lipids [8]. Therefore, it is likely BnCLIP1 is targeted toward not only the MCSs, but also other plastids in both photosynthetic and non-photosynthetic tissues.

### The transcript profiles of *BnCLIP1* during natural chloroplast senescence and chemical induced chloroplast disruption

As BnCLIP1 was detected at the MCSs between ER and chloroplasts (Fig. 3), it was meaningful to probe its role in chloroplast development and oil synthesis. Here, the transcript patterns of *BnCLIP1* in developing seeds at 25 DAP, 35 DAP, 45 DAP, and 50 DAP from *Brassica napus* lines EM91 and EM102 with low (29.62%) and high oil (50.59%) content, respectively, was investigated by real-time PCR. Figure 4C clearly shows that EM91 seeds were aging faster than EM102 seeds. When seeds developed up to 50 DAP, the seed capsules of EM91 turned black, while those of EM102 remained slightly green. We determined the seed chlorophyll content, including the content of chlorophyll *a* (Ca), chlorophyll *b* (Cb), and total chlorophyll *a* and *b* (Ca+b). We found that the seeds of both the varieties retained most of the chlorophyll at 35 DAP indicating they have the highest photosynthetic efficiency at 35 DAP (Fig. 4D). After 35 DAP, the chlorophyll content begins to decrease, and seeds start becoming senescent. Interestingly, during seed senescence, the transcript levels of *BnCLIP1* in both EM102 and EM91 were rapidly up-regulating (Fig. 4E). In addition, both the chlorophyll content and transcription level of *BnCLIP1* were much higher in the seeds of the high oil-content line (Fig. 4D, E).

Spectinomycin (SPCM) is a chloroplast-specific antibiotic, which can cause destruction of chloroplasts [47]. We treated *Brassica napus* leaves with SPCM to artificially cause leaf senescing and yellowing. Five-seconds per day treatment caused significant upregulation of *BnCLIP1* transcription, and 1-hour *in vitro* induction elevated the transcription level of BnCLIP1 for five folds (Fig. 4B).

## Discussion

In this study, the putative lipase gene, designated as *BnCLIP1* (GeneBankGenBank Acc. No. FJ461591) from *Brassica napus*, was homologous to the putative TAG lipase gene from *A. thaliana* (*At1g06800*). Based on bioinformatics analysis, the predicted protein BnCLIP1 was a chloroplast localized protein containing a plastid transit peptide, and belongs to the lipase class 3 family (Fig. 1). The truncated BnCLIP1 without the predicted signal peptide was exogenously expressed in the *S. cerevisia* (*pep4*). Total proteins extracted from the transformants were used for enzyme activity assay using p-nitrophenyl laurate (C12) as the substrate. However, no obvious enzyme activity of BnCLIP1 was detected towards this C12 substrate (p>0.05; data not shown). One of the reasons could be that p-nitrophenyl laurate is not the suitable substrate. This may also suggest BnCLIP1 may have quite different substrate spectrum from other lipases. But the Sudan black B staining, 2-D TLC and gas chromatography-mass spectrometry (GC-MS) results revealed that BnCLIP1 can decrease the cellular content of both neutral and polar lipids in yeast, and also has a substrate preference for C16:0 lipids (Fig. 2 and Table 1). Thus, we considered BnCLIP1 as a lipase with the substrate specificity.

Lipid metabolism is critical in the life cycle. Lipases were known to be involved in many physiological processes, such as converting phosphatidylcholine to substrate for galactolipid synthesis in chloroplast envelope [48], mediating the onset of senescence [49], providing carbon source and energy for seed germination [50], maintaining the structural integrity of chloroplast [4]. In plants, fatty acids are usually generated via the following two pathways, the degradation and synthesis pathway. In the degradation pathway, fatty acids from storage are transported to the glyoxysome and converted to acyl-CoAs for subsequent catalysis by β-oxidation [51]. In the synthesis pathway, fatty acids are synthesized in the plastids (mainly in the chloroplasts) by sucrose mobilization and photosynthesis [52]. The synthesized fatty acids have to be exported from plastid. The fatty acids released from each pathway are assembled into glycerol lipids in the ER [52]. In plants, the synthesis of the chloroplast galgacto-and sulfo-lipids in the chloroplast envelope requires the substrates to be trafficked from the ER [53], [54], [55], [56]. Membrane contact sites between ER and chloroplast have been shown to be the place where this trafficking occurs [19]. Moreover, the trafficking is often accompanied by lipids interconversion [29], [30], [31]. Lipid interconversion also occurs for other lipids, such as phospholipids, which can be replaced by DGDG in plasma membranes [26], [27], mitochondria [28] and tonoplasts [26] under phosphate starvation conditions. All these previous findings suggested that lipase might be required during lipids interconversion.

In this work, the *Brasscia napus* originated lipase BnCLIP1 was found to localize at the MCSs sites between ER and chloroplast in transient expressed tobacco leaves (Fig. 3), whereas not localized in plasma membrane (Fig. S1). As *BnCLIP1* mRNA was detected in non-photosynthetic tissues (Fig. 4A), and its eGFP fused protein was also found scattering at some unknown places around the ER and plasma membrane (Fig. 3 and Fig. S1), we suspected that BnCLIP1 is also targeting to other organelles, which requires further study. To probe the physiological function of BnCLIP1, we investigated its transcript patterns during the seed developing process for two *Brassica napus* lines with different oil-content. The result showed that BnCLIP1 was transcribed at different levels during the developing process of oil seeds. *BnCLIP1* was rapidly up-regulated in both lines at the maturation stage, during which period seeds were in senescence. This observation suggests BnCLIP1 may involve in chloroplast degradation. Interestingly, the senescence of seeds with high oil-content was started later than those with low oil-content (Fig. 4C, D), but the transcription response of BnCLIP1 in the former seeds was faster than the later ones suggesting a potential role of BnCLIP1 in affecting seed oil synthesis by regulating chloroplast integrity, since fatty acids are mainly synthesized in chloroplast [52]. We also investigated the transcript patterns of BnCLIP1 in specinomycin treated young leaves of *Brassica napus*. The result showed that BnCLIP1 is significantly up-regulated during the breakdown of chloroplast caused by specinomycin. Taken together, these results suggest that BnCLIP1 is involved in chloroplast degradation or maintaining during senescence by mobilizing lipids at the MCSs between ER and chloroplast. Although a lipase was observed to locate at the MCSs between ER and chloroplast in this study, there are still several interesting questions to be answered. Such as, whether the substrates of this lipase are from ER or chloroplast? And, whether the released fatty acids will go to chloroplast or ER? Further study is on the way.

## Supporting Information

Figure S1 **BnCLIP1-eGFP is not localized at the plasma membrane in transgenic tobacco leaves.** (A) The subcellular location of plasma membrane relative to chloroplast. (B) The subcellular location of BnCLIP1-eGFP relative to chloroplast. (C) The subcellular location of BnCLIP1-eGFP relative to plasma membrane. Chloroplast was shown in red autofluorescence; plasma membrane marked with red fluorescence protein (RFP) was colored red or green for obtaining clear overlapped spots with green or red fluorescence; while the cell counter was shown in the white background. The arrow head 5 indicates the scattered GFP signal. Scale bars:10ìm.
(TIF)
