# Supplementary figures and images for: A Brassica napus Lipase Locates at the Membrane Contact Sites Involved in Chloroplast Development
Source: PLoS One. 2011 Oct 26;6(10):e26831. doi: 10.1371/journal.pone.0026831 (PMC3202582; doi:10.1371/journal.pone.0026831)

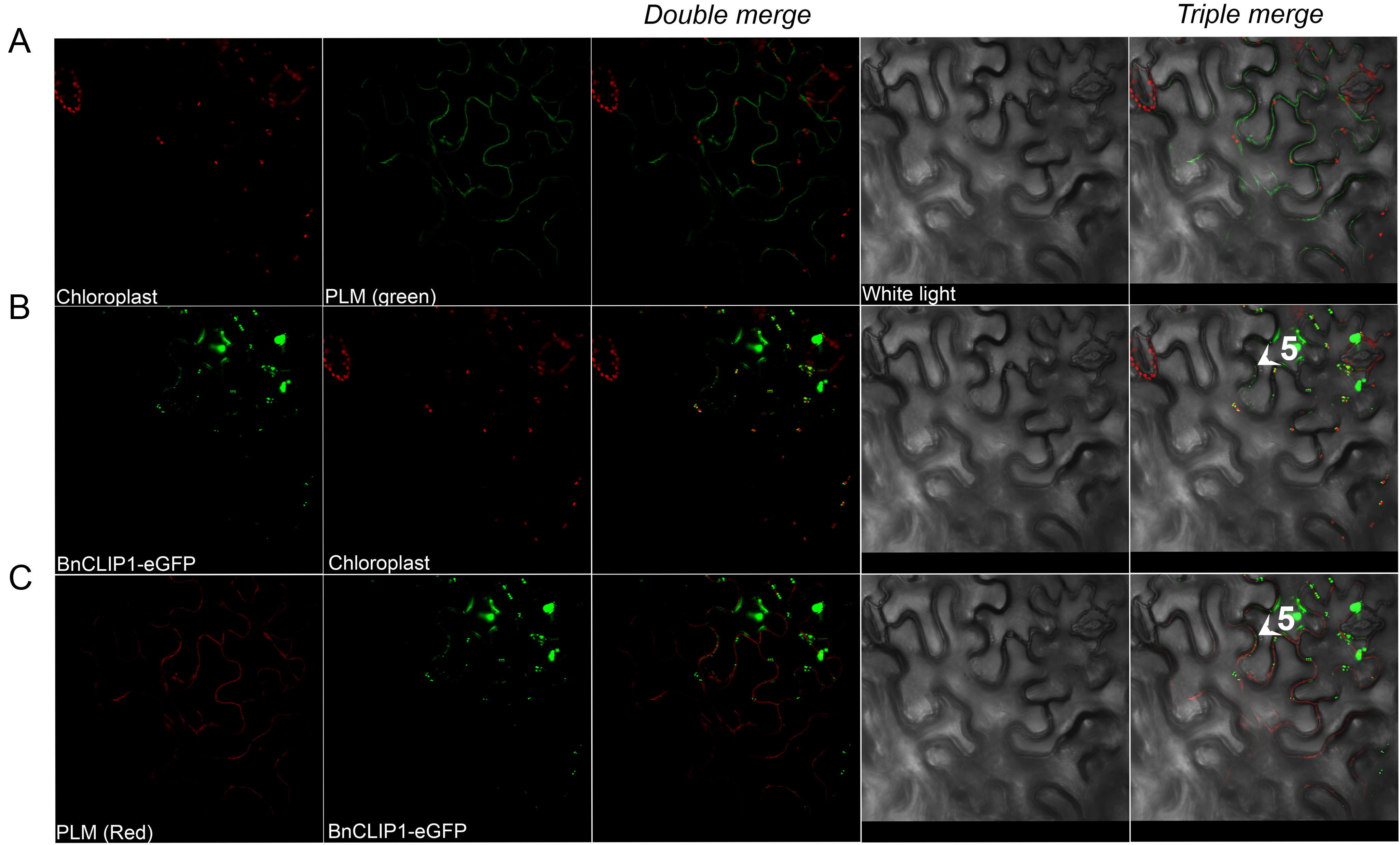

Supplement: Figure S1 — BnCLIP1-eGFP is not localized at the plasma membrane in transgenic tobacco leaves. (A) The subcellular location of plasma membrane relative to chloroplast. (B) The subcellular location of BnCLIP1-eGFP relative to chloroplast. (C) The subcellular location of BnCLIP1-eGFP relative to plasma membrane. Chloroplast was shown in red autofluorescence; plasma membrane marked with red fluorescence protein (RFP) was colored red or green for obtaining clear overlapped spots with green or red fluorescence; while the cell counter was shown in the white background. The arrow head 5 indicates the scattered GFP signal. Scale bars:10ìm. (TIF) [file pone.0026831.s001.tif]
